# Development and Investigation of an Innovative 3D Biohybrid Based on Collagen and Silk Sericin Enriched with Flavonoids for Potential Wound Healing Applications

**DOI:** 10.3390/polym16121627

**Published:** 2024-06-08

**Authors:** Lea Sleiman, Andreea-Daniela Lazăr (Popa), Mădălina Albu-Kaya, Minodora Maria Marin, Durmuș Alpaslan Kaya, Otilia-Ruxandra Vasile, Sorina Dinescu

**Affiliations:** 1 Department of Biochemistry and Molecular Biology, University of Bucharest, 050095 Bucharest, Romania; sleiman.lea@s.bio.unibuc.ro (L.S.); andreea.lazar@bio.unibuc.ro (A.-D.L.); 2 The National Research and Development Institute for Textiles and Leather (INCDTP)-Division Leather and Footwear Research Institute, 93 Ion Minulescu Str., 031215 Bucharest, Romania; albu_mada@yahoo.com; 3 Advanced Polymer Materials Group, Faculty of Applied Chemistry and Materials Science, Politehnica University of Bucharest, 1-7 Polizu Street, 01106 Bucharest, Romania; maria_minodora.marin@upb.ro; 4 Department of Field Crops, Faculty of Agriculture, Hatay Mustafa Kemal University, Antakya-Hatay 31034, Turkey; dak1976@msn.com; 5 Science and Engineering of Oxide Materials and Nanomaterials Department, Faculty of Chemical Engineering and Biotechnologies, Politehnica University of Bucharest, 1-7 Polizu Street, 01106 Bucharest, Romania; otilia.vasile@upb.ro; 6 Research Institute of the University of Bucharest (ICUB), 050663 Bucharest, Romania

**Keywords:** wound healing, biomaterials, curcumin, quercetin, collagen, silk sericin, tissue regeneration

## Abstract

Skin tissue injuries necessitate particular care due to associated complex healing mechanisms. Current investigations in the domain of tissue engineering and regenerative medicine are focused on obtaining novel scaffolds adapted as potential delivery systems to restore lost tissue functions and properties. In this study, we describe the fabrication and evaluation of a novel 3D scaffold structure based on collagen and silk sericin (CollSS) enriched with microcapsules containing natural compounds, curcumin (C), and/or quercetin (Q). These 3D composites were characterized by FT-IR spectroscopy, water uptake, in vitro collagenase degradation, and SEM microscopy. Furthermore, they were biologically evaluated in terms of biocompatibility, cell adhesion, anti-inflammatory, and antioxidant properties. All tested materials indicated an overall suitable biocompatibility, with the best results obtained for the one containing both flavonoids. This study suggests the cumulative beneficial effect of C and Q, encapsulated in the same composite, as a potential non-invasive therapeutic strategy for skin tissue regeneration in patients suffering from chronic wounds.

## 1. Introduction

Treatment of chronic wounds represents one of the most challenging approaches in the domain of tissue engineering and regenerative medicine due to the complex nature of associated healing mechanisms. Wound healing constitutes a dynamic and intricate physiological process comprised of three primary overlapping phases: (i) inflammatory phase (days 0–4) characterized by the initiation of homeostasis and release of an array of bioactive molecules including several growth factors and cytokines such as fibroblast growth factor (FGF), epidermal growth factor (EGF), interleukin 6 (IL-6), and tumor necrosis factor (TNF), in addition to reactive oxygen species (ROS); (ii) proliferation phase (days 5–20) characterized by epithelial cells migration, fibroblast cells proliferation and establishment of “granulation tissue”; and (iii) tissue remodeling phase (months–years) signified by lesion contraction and reconstruction of extracellular matrix, primarily through the deposition of collagen type I [1]. 

Different treatment approaches have focused on developing biomaterials that can serve as wound dressings to enhance wound healing and skin regeneration. Multiple studies have shown that these materials can provide a 3D in vivo-like environment to promote cell growth and development [2,3]. Biomaterials can be made from either natural polymers (e.g., collagen, sericin, chitosan, alginate, etc.), synthetic polymers (e.g., polyacrylic acid, poly(N-isopropylacrylamide), polylactic acid, poly-ε-caprolactone, etc.) or from a combination of both. 

Natural polymers are widely used in the formulation of wound dressings due to their biocompatible, antioxidant, and anti-inflammatory properties [4,5]. Collagen presents high tensile strength, elasticity, low antigenicity, and suitable biocompatibility and can function in wound healing by promoting fibroblast proliferation and wound contraction [6,7]. However, biomaterials based on pure collagen may not be ideal for wound dressings due to low biostability and limited mechanical resistance; for that reason, crosslinking collagen with other natural polymers can help promote skin regeneration and wound healing [8]. Sericin, also known as sticky protein, is one major protein from silk used in numerous tissue engineering studies. It has been shown to promote cell proliferation and adhesion to the substrate [9]. This natural polymer, in association with collagen, can be added to various biomaterials to enhance cell–scaffold interaction [10,11,12].

Current investigations in the domain of tissue engineering and regenerative medicine are focusing on the development of advanced biomaterials designed to serve as delivery systems. Natural compounds, such as flavonoids, may be integrated within their matrix architecture, hence providing a controlled and localized drug release. Curcumin and quercetin are bioactive flavonoids widely used for wound regeneration and healing purposes. Several studies have proven that these compounds present strong antioxidant, anti-inflammatory, and immuno-modulatory properties [13,14,15]. However, their therapeutic application is limited due to their insolubility and poor availability. These limitations can be addressed through the development of novel delivery systems designed to improve the localized absorption of these natural compounds at the sight of injury. Such systems can be represented by biocompatible materials incorporating polymeric microcapsules. These design principles may be helpful in promoting a positive patient response to novel therapeutic strategies by overcoming the limits and risks associated with systemic administration [16]. 

In this context, we investigated the effect of innovative microcapsules enriched with natural compounds, C and/or Q, incorporated into a scaffold structure based on collagen and sericin (CollSS). The biological properties of these 3D materials were evaluated in terms of biocompatibility, cell adhesion, anti-inflammatory, and antioxidant effects.

## 2. Materials and Methods

Type I fibrillar collagen (Coll) in gel form, with an initial concentration of 2.43% and pH 3, high degree purity, and triple helical structure, was obtained using the current technology [17] available in the Collagen Department of INCDTP. Silk sericin (SS, isolated from *Bombyx mori*) and alginic acid sodium salt from brown algae (Alg) were purchased from Sigma-Aldrich (Darmstadt, Germany), gelatin and sodium carboxymethyl cellulose (CMCNa) from Fluka (Darmstadt, Germany), while curcumin (C) from Alfa Aesar (Heysham, UK) and quercetin (Q) was obtained from Thermo Scientific (Kandel, Germany). Calcium chloride (CaCl_2_) and glutaraldehyde from Merck (Hohenbrunn, Germany), were used as crosslinking agents. Sodium hydroxide (Merck, Darmstadt, Germany) was of analytical grade, and the water was distilled.

### 2.1. Microcapsules Preparation

The microcapsules were prepared using the previously described protocol [18]. Briefly, Alg/CMCNa/gelatin with a ratio of 4:1:10 was solubilized in distilled water, together with 0.08% C and/or 0.04% Q, and this mixture was embedded as a natural active substance. The obtained gel was dropped by a syringe into a solution of CaCl_2_ for crosslinking, resulting in microcapsules with C and/or Q.

### 2.2. CollSS-Based Scaffolds Preparation

The collagen gel was adjusted at a concentration of 1.5% and physiological pH (7.4) using 0.1 M NaOH. A ratio of 100:40 collagen to sericin represented the control sample (CollSS), and 30% microcapsules with either C, Q, or a mixture of these flavonoids (CQ) were added, as Table S1 presents, in order to obtain the other compositions. All the gels were crosslinked with glutaraldehyde (0.1%) and then freeze-dried following a 48 h program as shown in Figure S1. 

Freeze-drying is a low-temperature dehydration process that involves sublimation under vacuum (transition from solid to vapor state) and has been carried out for 48 h, as shown in the diagram in Figure S1.

After freeze-drying, CollSS spongious scaffolds with or without C and/or Q microcapsules were obtained. 

### 2.3. The FT-IR Analysis for CollSS-Based Scaffolds

Using an attenuated total reflectance (ATR) accessory on a Vertex 70 Bruker FT-IR spectrometer (Billerica, MA, USA), measurements of Fourier-Transform Infrared Spectroscopy (FT-IR) were carried out. The FT-IR spectra were registered in the 600–4000 cm^−1^ wavenumber range at a resolution of 4 cm^−1^ using the ATR-FT-IR mode for every formulation that was manufactured.

### 2.4. The Water Uptake Analysis for CollSS-Based Scaffolds

The water uptake capacity was investigated using the protocol described in our previous studies [19]. Briefly, 1 cm^2^ from each sample was weighed before and after immersion, and the ability to keep water in the porous structures was registered at different time points. 

### 2.5. In Vitro Degradation of CollSS-Based Scaffolds

CollSS-based scaffolds were in vitro degraded using collagenase solution (1 μg/mL) in PBS at 37 °C according to the previously described method [20]. The mass lost in time was calculated for all samples in triplicate.

### 2.6. Scanning Electron Microscopy for CollSS-Based Scaffolds

The scanning electron microscopy (SEM) studies on CollSS samples were performed on a Hitachi S4500 system (Hitachi High-Technologies Europe GmbH, Krefeld, Germany) to determine the form and size of the spongious structure.

### 2.7. In Vitro Study of Material Biocompatibility

The biological effect of the various materials (CollSS, CollSS-C, CollSS-Q, and CollSS-CQ) supplemented or not with phytocompounds was evaluated by performing a series of biocompatibility tests. Normal skin keratinocytes (HaCaT, code 300493, CLS) were cultured in Dulbecco’s Modified Eagle Medium (DMEM) supplemented with 10% Fetal Bovine Serum (FBS) and 1% antibiotic antimycotic solution (Sigma Aldrich, Darmstadt, Germany) and were maintained in standard culturing conditions (37 °C, 5% CO_2_, humidity). The materials were sterilized by UV light exposure, washed with serum-free medium and 5% antibiotic to remove undesirable toxic products, and then cut into discs of 1 cm^2^ diameter. Afterward, cells were seeded at a density of 2.5 × 10^5^ cells/cm^2^ and allowed to distribute inside the 3D materials under standard culture conditions, during which the biocompatibility of the materials was assessed through quantitative (MTT, LDH) and qualitative (Live/Dead fluorescence microscopy) tests, at three and seven days after seeding. Cell adhesion assay was carried out at 48 h post-seeding.

### 2.8. Quantitative and Qualitative Assessment of Cell Viability, Proliferation, and Cytotoxicity

Cell viability and proliferation of keratinocytes in contact with the materials were assessed by MTT assay (methylthiazolyldiphenyl tetrazolium bromide, Sigma-Aldrich, Steinheim, Germany). The solution was prepared in PBS at a concentration of 1 mg/mL according to the manufacturer’s instructions. The samples were incubated for four hours with MTT solution at 37 °C to allow the crystallization of formazan. Isopropanol was used to dissolve the obtained formazan crystals, and the solution’s absorbance was measured at 550 nm using a FlexStation 3 Spectrophotometer (Molecular Devices, San Jose, CA, USA), thus indicating the number of metabolically active cells.

The cytotoxic effect of the materials was evaluated by LDH assay (TOX7-1KT, Sigma-Aldrich, Steinheim, Germany). The LDH reaction mix was prepared according to the manufacturer’s guidelines and was added to the collected cell media in a 96-well plate. The samples were incubated in the dark for 20 min at room temperature. The lactate dehydrogenase released in the culture medium by the cells that lost their membrane integrity was quantified spectrophotometrically, with the absorbance of the final product measured at 490 nm using a FlexStation 3 Spectrophotometer (Molecular Devices, San Jose, CA, USA). 

The Live/Dead test was used to evaluate the behavior and viability of keratinocytes put in contact with the materials. The assay was performed using the Live/Dead kit (ThermoFisher Scientific, Waltham, MA, USA) by labeling live cells (green) with calcein acetoxymethyl (AM) and nuclei of dead cells (red) with ethidium homodimer-1 (EthD-1). The solution was prepared according to the manufacturer’s instructions and added to the samples, and then the plate was incubated in the dark for 1 h and 30 min at room temperature. Cells were visualized using a laser-scanning confocal microscope (Nikon A1\/A1R Confocal Laser Microscope System, Nikon Instruments Inc., Melville, NY, USA). The images were analyzed using the NIS-Elements software (Nikon Instruments Inc., version 5.11 64-bit, Melville, NY, USA). 

### 2.9. Cytoskeleton Development of Keratinocytes in Contact with the Biomaterials

The adhesion of keratinocytes to the 3D materials was investigated after 48 h of incubation. Cells were fixed with 4% paraformaldehyde (PFA) solution (Sigma Aldrich Co., Steinheim, Germany) made in PBS for 1 h and 30 min at 4 °C. Next, the cells were permeabilized with 0.1% Triton X (Sigma Aldrich Co., Steinheim, Germany) and 2% bovine serum albumin (BSA) solution prepared in PBS for 1 h at 4 °C. This step was followed by the addition of phalloidin-FITC (Sigma Aldrich Co., Steinheim, Germany) solution for 1 h in the dark at room temperature to allow the staining of the F-actin filaments. Nuclei labeling was performed with Hoechst 33342 (ThermoFisher Scientific, Foster City, CA, USA) solution for 15 min in the dark, at room temperature. Cell visualization was performed utilizing a laser-scanning confocal microscope (Nikon A1/A1R Confocal Laser Microscope System, Nikon Instruments Inc., Melville, NY, USA). The images were analyzed using the NIS-Elements software (Nikon Instruments Inc., version 5.11 64-bit, Melville, NY, USA).

### 2.10. Gene Expression Evaluation of Apoptotic Markers by qPCR

To investigate the effect on cell survival of the materials supplemented with flavonoids, especially those enriched with both curcumin and quercetin, the gene expression of markers involved in apoptosis (*Bax*, *Bcl-2*, and *Caspase-3*) was evaluated by qPCR. Total RNA from HaCaT cells was isolated using the classical TRIzol-based method (ThermoFisher Scientific, Waltham, MA, USA) following the manufacturer’s guidelines. RNA concentration and purity were assessed by spectrophotometric analysis (NanoDrop 8000 spectrophotometer, Thermo Scientific, Waltham, MA, USA). Subsequently, the RNA samples were reverse transcribed to the corresponding cDNA using the iScript cDNA Synthesis kit (Bio-Rad, Hercules, CA, USA) and the Veriti 96-Well Thermal Cycler (Applied Biosystems, Waltham, MA, USA). Sequences of gene-specific primers used in qPCR assay are presented in Table S2. Gene expression of these apoptotic markers was assessed by qPCR using the 2x Forget-Me-Not qPCR kit and Viia7 Real-Time PCR system (Thermo Scientific, Waltham, MA, USA). The tested samples were normalized to the reference gene GAPDH.

### 2.11. Protein Expression Evaluation of Apoptotic Markers by Confocal Microscopy

Protein expression evaluation of pro-apoptotic marker Bax and anti-apoptotic marker Bcl-2 in keratinocytes exposed to CollSS-based scaffolds was performed using immunofluorescence coupled with confocal microscopy. Cells were fixed with a solution of PFA 4% for 1 h and 30 min at 4 °C, after which cell permeabilization was carried out using a 2% BSA solution with 0.1% Triton X for 1 h at 4 °C. The cells were incubated overnight at 4 °C with primary antibody solutions (1:100 dilution for Bax mouse monoclonal antibody, SantaCruz Biotechnology, Inc., Dallas, TX, USA; 1:300 dilution for Bcl-2 rabbit polyclonal antibody, Proteintech, Planegg-Martinsried, Germany). Subsequently, secondary antibody solutions were added (1:500 dilution for AF488-labeled goat anti-mouse IgG and for AF546-labeled goat-anti-rabbit IgG, SantaCruz Biotechnology, Inc., Dallas, TX, USA) for 2 h in the dark at room temperature. The nuclei were stained with Hoechst 33342 solution for 15 min in the dark at room temperature. The cells were visualized under a laser-scanning confocal microscope (Nikon A1/A1R Confocal Laser Microscope System). The images were analyzed using the NIS-Elements software (Nikon Instruments Inc., version 5.11 64-bit, Melville, NY, USA).

### 2.12. Fluorescence Quantification of Cell Imaging 

Quantification of fluorescence intensity for Live/Dead images, as well as protein expression of apoptotic markers, was performed with ImageJ software (version 1.x bundled with 64-bit Java 8), while for graphical representation, the obtained data were analyzed using GraphPad Prism software, version 9.0 (GraphPad Software Inc., San Diego, CA, USA). The statistical validity of the results was assessed using the one-way ANOVA method and Bonferroni correction post-test. Data are expressed as mean ± SD for n = 10 fields of view/sample; *p* < 0.05 was considered statistically significant.

### 2.13. Evaluation of Anti-Inflammatory Potential of CollSS-CQ Biomaterial

A culture of murine macrophages from the RAW 264.7 cell line was maintained under standard conditions to evaluate the anti-inflammatory potential of the CollSS-CQ biohybrid. Cells were activated with a 1 μg/mL lipopolysaccharide (LPS) solution (Sigma-Aldrich, Darmstadt, Germany) 24 h prior to seeding. Both gene and protein expression of pro-inflammatory cytokines IL-6 and TNF-α was assessed 48 h after seeding, with the simple CollSS material devoid of flavonoids used as control. A positive control of plastic-seeded activated macrophages exposed to no treatment was also used as a reference for analysis. Sequences of gene-specific primers used in qPCR assay are presented in Table S2. Protein expression was analyzed qualitatively through immunofluorescence coupled with confocal microscopy using the protocol described in Section 2.11. (primary antibody solutions were diluted 1:300 and purchased from Proteintech, Planegg-Martinsried, Germany). The protein levels of pro-inflammatory mediators IL-6 and TNF-α were also measured from cell culture media 48 h after induced macrophages were put in contact with the CollSS-based systems, using enzyme-linked immunosorbent assay (ELISA, Qiagen, Hilden, Germany) and detection at 450 nm using a FlexStation3 system (Molecular Devices, San Jose, CA, USA). Data were analyzed using GraphPad Prism software, version 9.0 (GraphPad Software Inc., San Diego, CA, USA).

### 2.14. Evaluation of Antioxidant Potential of CollSS-CQ Biomaterial

To evaluate the antioxidant properties of the composite enriched with both flavonoids, a culture of murine macrophages from the RAW 264.7 cell line was obtained in standard conditions. These cells were treated before seeding with a 400 µM solution of H_2_O_2_ for 2 h. After being put in contact with the CollSS and CollSS-CQ biomaterials, the levels of reactive oxygen species (ROS) were evaluated fluorimetrically at three different time points (24, 48, and 72 h post-seeding) using the Amplex^TM^ Red Hydrogen Peroxide/Peroxidase Assay Kit (10-acetyl-3,7-dihydroxyphenoxazine) (cat. No. A22188, ThermoFisher Scientific, Waltham, MA, USA). A control sample of peroxide-treated macrophages grown on plastic was considered a positive reference. Moreover, to further evaluate the antioxidant capacity of the materials, the DPPH Antioxidant Assay Kit (K2078, Abcam, Cambridge, UK), a high-throughput adaptable, microplate-based assay, which employs 2,2-diphenyl-1-picrylhydrazyl radical (DPPH), that acts as a scavenger for hydrogen radical, was used. The DPPH method produces a decrease in absorbance at 517 nm (FlexStation3 system, Molecular Devices, San Jose, CA, USA), proportional to the antioxidant capacity of the samples. The synthetic antioxidant Trolox is used to standardize the sample antioxidant capacity. The following formula was used to compute the percentage of antioxidants (RSA): % of antioxidant activity = [(Ac − As) ÷ Ac] × 100, where Ac—control reaction absorbance; As—testing specimen absorbance. Data were analyzed using GraphPad Prism software, version 9.0 (GraphPad Software Inc., San Diego, CA, USA).

### 2.15. Statistical Analysis

All the experiments were performed in triplicate (n = 3), and the obtained data were processed using the GraphPad Prism 6 software (GraphPad Software Inc., San Diego, CA, USA). The one-way ANOVA method and Bonferroni correction post-test were used to assess the statistical validity of the results, considered to be significant for *p* < 0.05.

## 3. Results

CollSS-based scaffolds were characterized by FT-IR spectroscopy, water uptake, in vitro collagenase degradation, and SEM microscopy.

### 3.1. The FT-IR Analysis for CollSS-Based Scaffolds 

**Figure 1:**
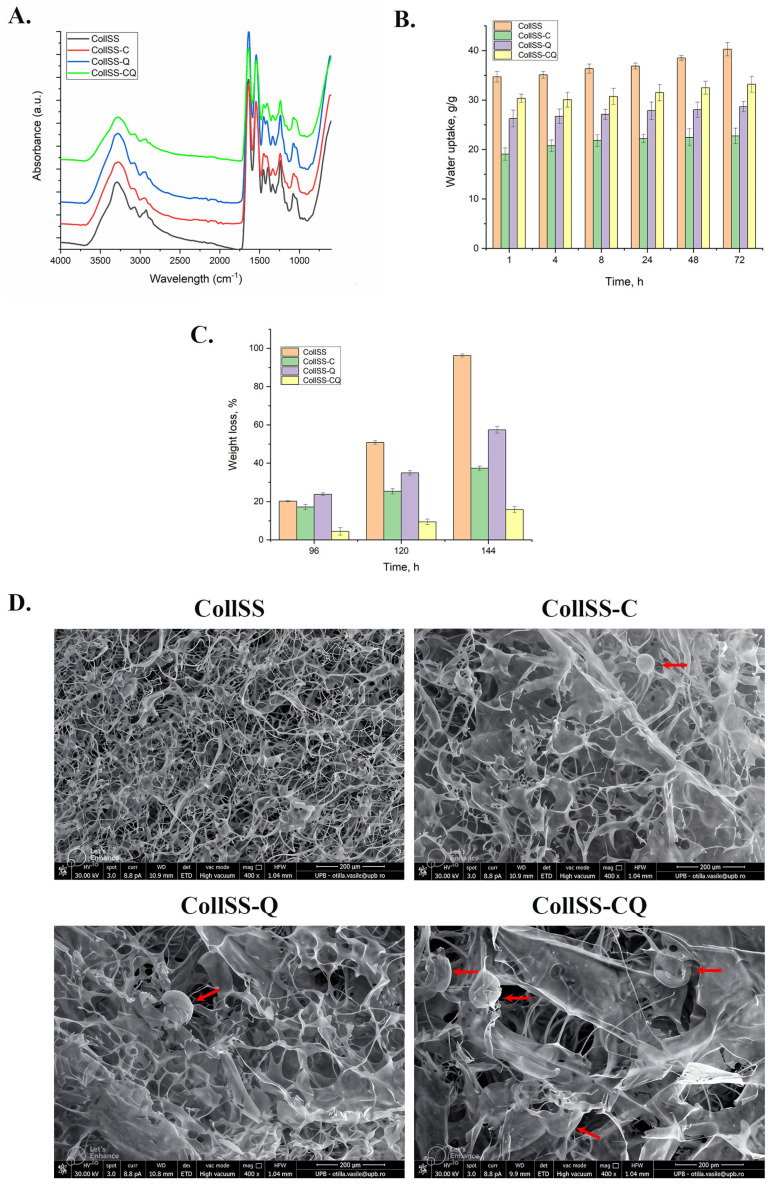
Biophysical parameters of CollSS−based scaffolds with C and/or Q microcapsules: (**A**) structural characterization by FT−IR spectroscopy; (**B**) water uptake analysis showcasing the hydrophilic nature of the materials; (**C**) in vitro degradation behavior in collagenase solution at physiological temperature and pH; (**D**) SEM images showcasing the porous structure of the CollSS−based scaffolds (×400 magnification). The red arrows highlight the microcapsules embedded within the materials.

The scaffolds with the compositions shown in Table S1 were characterized by the FT-IR technique (Figure 1A) to investigate the structural modifications of collagen at the level of secondary structure.

The spectra of the CollSS samples display the following specific protein-related peaks (collagen and sericin): the amide A band, which is located at approximately 3288 cm^−1^ and is correlated to the N—H stretch; the amide B band, which is located at approximately 2934 cm^−1^ and is related to the symmetric stretching of the CH_2_ groups; the amide I band, which can be observed at approximately 1633 cm^−1^ and is associated with the stretching vibration of the C=O (carbonyl) groups as well as the C—N group; the amide II band, which is assigned to N—H stretching vibrations, related to C—N and C—C stretching vibrations, is detected at approximately 1543 cm^−1^; the amide III band is located at approximately 1237 cm^−1^, being related to the vibrational opening of the C—N and N—H groups, as well as to the vibrations of the CH_2_ groups in the glycine support and secondary chains of proline [21,22,23,24,25]. 

### 3.2. The Water Uptake Analysis for CollSS-Based Scaffolds 

The CollSS-based scaffolds were designed to be used for wound healing, and one of their most important properties is hydrophilicity. The extent of water uptake (swelling properties) can provide information about the space available for tissue ingrowth [26]. 

As Figure 1B presents, all the samples absorb a very high amount of water starting from the first hour (between about 20 to 35 g/g) and continue to swell no more than 6% in 72 h. The most hydrophilic sample is the control one (CollSS), while the sample containing curcumin (CollSS-C) absorbs the smallest amount of water. 

### 3.3. In Vitro Degradation of CollSS-Based Scaffolds

In order to simulate the in vivo conditions, CollSS-based scaffolds were degraded in collagenase solution at physiological temperature and pH. It is well known that collagenase is the specific enzyme that degrades collagen down to amino acids. As can be seen from Figure 1C, the CollSS scaffold is degraded in a higher proportion (96%) over the tested time frame, while the most resistant one is shown to be CollSS-CQ, which contains microcapsules with both curcumin and quercetin.

### 3.4. Scanning Electron Microscopy for CollSS-Based Scaffolds 

The pore size and density of CollSS sponges play an important role in the effectiveness of cell infiltration into the interstitial space of the matrix. If the pores are too small, the cells cannot penetrate and remain on the surface of the scaffold, and if they are too large, the cells pass down. Figure 1D shows SEM images for the CollSS-based scaffolds. 

As observed in the SEM images, all structures, represented by CollSS scaffolds with and without microcapsules (C, Q, or CQ), display very high porosity with interconnected pores. The CollSS shows a dense and ordered structure, while the samples containing microcapsules (marked with red arrows) have a more well-defined porous structure, with pores formed between the lamellae and microcapsules interspersed. The pore sizes are between 50 and 100 μm for all CollSS-based scaffolds.

### 3.5. In Vitro Assessment of CollSS-Based Materials Biocompatibility and Cytoskeleton Development

The cellular behavior and response to the different materials supplemented or not with flavonoids (CollSS, CollSS-C, CollSS-Q, and CollSS-CQ) were evaluated through quantitative and qualitative tests. This allows an understanding of how the 3D architecture of the material influences cell adherence and survival. 

**Figure 2:**
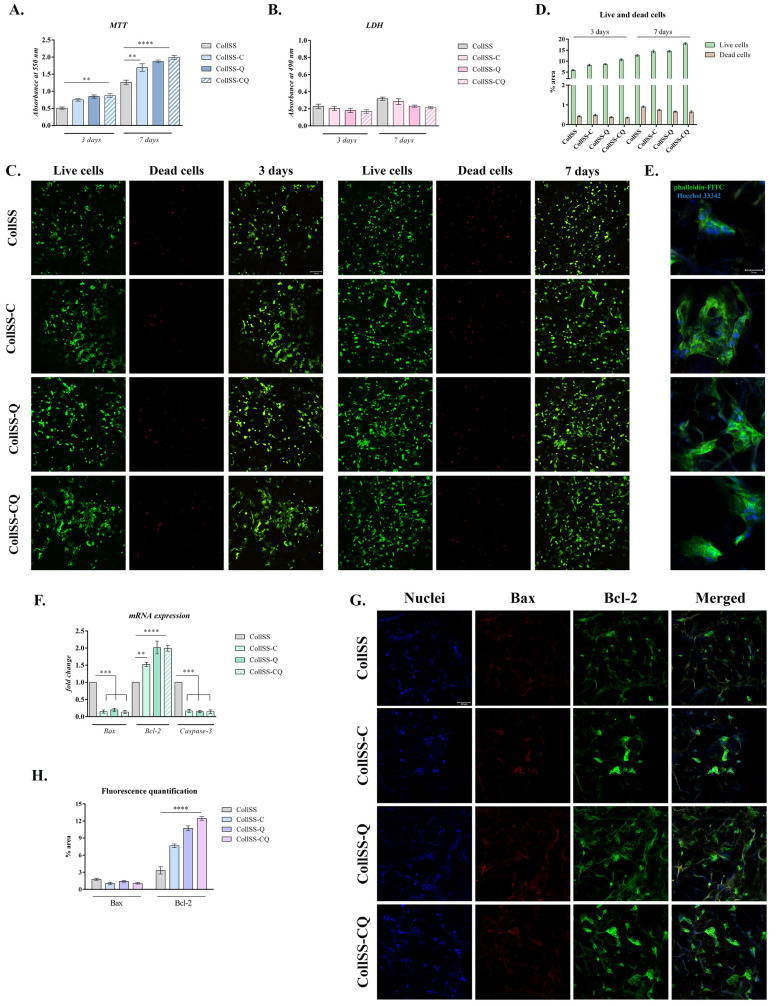
Biocompatibility profile of tested materials put in contact with HaCaT keratinocytes: (**A**) MTT assay performed to highlight cell viability and proliferation. (**B**) LDH assay performed to evaluate the cytotoxicity of the materials. (**C**) Confocal microscopy images of live cells (green) and nuclei of dead cells (red) visualized after 3 and 7 days of culture in CollSS, CollSS-C, CollSS-Q, and CollSS-CQ biosystems (scale: 100 µm). (**D**) Quantification of green fluorescence (live cells) levels and red fluorescence (dead cell nuclei) levels in all composites. (**E**) Cytoskeleton assessment of HaCaT in contact with CollSS-based biomaterials after 48 h of seeding under standard conditions. F-actin filaments are labeled with phalloidin-FITC (green) and cell nuclei with Hoechst 33342 (blue). Scale: 20 µm. (**F**) Gene expression profile of pro-apoptotic markers Bax and Caspase-3 and the anti-apoptotic marker Bcl-2, compared between the tested materials (CollSS, CollSS-C, CollSS-Q, and CollSS-CQ). Data are normalized to GAPDH expression level. (**G**) Pro-apoptotic marker Bax and anti-apoptotic marker Bcl-2 protein expression in keratinocytes exposed to CollSS-based scaffolds (scale: 50 µm). (**H**) Quantification of green fluorescence (protein expression of Bcl-2, labeled AF488) levels and red fluorescence (protein expression of Bax, labeled AF546) levels on all composites. Statistical significance: *p* < 0.01 (**), *p* < 0.001 (***), and *p* < 0.0001 (****).

Results obtained from quantitative cell viability assessment showed that the tested materials enriched with C and/or Q microcapsules displayed overall suitable biocompatibility. Three days after cell seeding, statistically significant differences in cell viability were registered among the four tested materials. The curcumin-enriched CollSS scaffold increased cell viability when compared to the CollSS material used as a control. Similar cell viability was found for CollSS-Q and CollSS-CQ, with an increase of approximately twofold (*p* < 0.01), as compared to CollSS (Figure 2A).

Seven days after cells were put in contact with the materials, a significant increase in the level of cell metabolic activity (Figure 2A) was observed at the level of all composites, especially those enriched with flavonoids (*p* < 0.01 for CollSS-C and *p* < 0.001 for CollSS-Q and CollSS-CQ), hence proving that the addition of natural flavonoids in the composition of the tested materials positively influences the biocompatibility of the overall system. Interestingly, it is noted that the CollSS composite supplemented with both flavonoids C and Q had the highest cell viability rate and proliferation when compared to the other composites supplemented with only one of the flavonoids. Thus, it is suggested that C and Q display cumulative activity, favoring cell viability and proliferation. 

The cytotoxic effect of the composites was evaluated through LDH quantitative assay. The data obtained after 3 days of culture revealed low cytotoxicity, with similar levels for all tested biomaterials (Figure 2B). After 7 days, a slight increase in LDH levels was observed in all tested composites; however, this increase is statistically insignificant. The obtained results indicate the overall low cytotoxicity and suitable biocompatibility of the CollSS scaffolds enriched with C and/or Q.

Qualitative data obtained from the Live/Dead assay coupled with confocal microscopy allowed the visualization of live cells (marked in green) and nuclei of dead cells (marked in red) after 3 and 7 days, respectively (Figure 2C). Microscopy images show increased cell viability and proliferation 3 days after cell seeding, indicating that all composites support cell viability and growth. These results confirm the quantitative data obtained from MTT and LDH assays. After 7 days, a higher proliferation rate was observed at the level of all composites, especially those enriched with C and/or Q, when compared to the simple control. Notably, higher proliferation and a significant number of live cells were found in the case of CollSS-CQ material between days 3 and 7 of culture in standard conditions. This suggests that the dual incorporation of C and Q in collagen–sericin-based scaffolds supports cell survival and proliferation, suggesting favorable properties for wound healing capacities. Quantification of green fluorescence (live cells) levels and red fluorescence (dead cell nuclei) levels in all composites corroborated the qualitative results obtained through microscopy images, with a much higher amount of green fluorescence (5–15× higher) than red fluorescence (Figure 2D). 

The F-actin microfilaments were marked with phalloidin-FITC (green) to investigate the cytoskeleton properties of keratinocytes in contact with the materials. Images obtained from confocal microscopy (Figure 2E) show suitable cell adhesion to the substrate, as compared to the CollSS composite not supplemented with flavonoids, used as control. A well-defined cytoskeleton with organized distribution of elongated actin filaments can be observed, suggesting the positive changes occurring at the level of cell morphology and cytoskeletal architecture. Moreover, the obtained images reveal the adoption of an elongated, spindle-shaped cell morphology compared to the control. Thus, the enrichment of CollSS-based biohybrids with both C and Q provides the optimal condition to ensure cellular adhesion and suitable interaction with the substrate, properties that are essential for potential use in wound healing applications.

### 3.6. Evaluation of Apoptotic Markers Gene Expression by qPCR

To further investigate the ability of the CollSS materials supplemented with flavonoids to promote cell survival, the gene expression of markers involved in apoptosis (*Bcl-2*, *Bax*, and *Caspase-3*) was assessed by qPCR in HaCaT cells, taking the material devoid of phytocompounds as control (Figure 2F). The obtained data indicate that at the level of CollSS composites enriched with C and/or Q, the gene expression of the anti-apoptotic marker *Bcl-2* showed a significant increase when compared to the expression of the pro-apoptotic markers *Bax* and *Caspase-3,* which registered a significant decrease. Moreover, for the materials supplemented with flavonoids, an increase of two folds in the expression of *Bcl-2* was observed when compared to the control (*p* < 0.01 for CollSS-C, *p* < 0.0001 for CollSS-Q and CollSS-CQ) (Figure 2F). Similar results were obtained at the protein level (Figure 2G), with a higher expression of Bax on the materials enriched with flavonoids, whereas Bcl-2 was poorly expressed in all tested composites. Quantification of fluorescence level (Figure 2H) showcased a significant difference for Bax protein expression, with an increase of more than 10× for the composite with both C and Q when compared to the control. These data suggest that the addition of C and/or Q microcapsules in the composition of the materials favors cell survival through an anti-apoptotic effect. 

Based on the results obtained from the previous analysis (cell viability and proliferation, cytotoxicity, cytoskeleton development), the CollSS-CQ composite was selected for further biological testing. 

### 3.7. Analysis of Anti-Inflammatory Potential for CollSS-CQ

**Figure 3:**
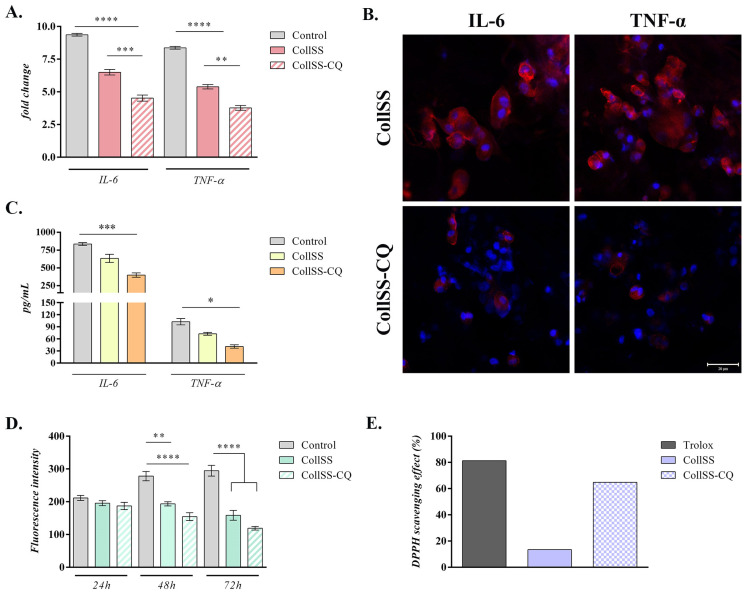
Anti-inflammatory and antioxidant activity of CollSS-based scaffolds: (**A**) TNF-α and IL-6 gene expression in activated macrophages exposed to CollSS and CollSS-CQ, as compared to the activated control. Data are normalized to GAPDH expression level. (**B**) Protein levels of TNF-α and IL-6 pro-inflammatory mediators found in cell culture media of activated macrophages after contact with the CollSS-based systems, as compared to the positive control. (**C**) TNF-α and IL-6 protein expression in activated macrophages exposed to the scaffolds (scale: 20 µm). (**D**) Colorimetric analysis of ROS production levels in cell culture media of activated macrophages after contact with the tested materials (CollSS and CollSS-CQ), compared to the positive control. (**E**) DPPH radical scavenging activity (RSA, %) of the tested composites, compared to standard Trolox. Statistical significance: *p <* 0.05 (*), *p* < 0.01 (**), *p* < 0.001 (***), and *p* < 0.0001 (****).

To evaluate the anti-inflammatory potential of the CollSS-CQ biohybrid, the expression of pro-inflammatory cytokines IL-6 and TNF-α was determined at gene and protein levels (Figure 3). A statistically significant decrease in the mRNA levels of IL-6 and TNF-α was registered in cells found in contact with CollSS-CQ and even CollSS, compared to the positive control (*p* < 0.0001). Notably, the most significant changes were detected for the collagen and sericin-based composite enriched with both flavonoids, where the expression of the pro-inflammatory markers was significantly downregulated, compared to the simple material (*p* < 0.001 for IL-6 and *p* < 0.01 for TNF-α), suggesting the enhanced anti-inflammatory effect of the biohybrid enriched with curcumin and quercetin (Figure 3A). 

To further validate the obtained results, IL-6 and TNF-α protein expression was assessed by immunostaining coupled with fluorescent microscopy (Figure 3B). Confocal microscopy images showed a significant decrease in IL-6 and TNF-α expression in macrophages exposed to the CollSS-CQ biohybrid, compared to the ones seeded on CollSS, suggesting that the encapsulated phytocompounds, C and Q, have a strong anti-inflammatory effect. Additionally, a clear decrease in pro-inflammatory cytokine levels secreted by cells in contact with CollSS-CQ (*p* < 0.001), as compared to CollSS and the positive control, was also observed through ELISA assay (Figure 3C), in accordance with the aforementioned results, and indicating the improved anti-inflammatory response of CollSS-CQ. 

### 3.8. Analysis of Antioxidant Potential of CollSS-CQ

The antioxidant potential of the CollSS-CQ biohybrid was evaluated by performing a colorimetric analysis to measure the production levels of reactive oxygen species (ROS) (Figure 3D), as well as a DPPH radical scavenging activity (RSA) assay for all tested composites (Figure 3E). After 24 h of cell culture in standard conditions, ROS secretion in the tested materials remained approximately constant with no statistically significant differences. However, after 48 h, a statistically significant decrease in ROS release was detected in the two composites, being much more prominent in the case of the material enriched with flavonoids (*p* < 0.01 for CollSS and *p* < 0.0001 for CollSS-CQ, respectively), compared to the positive control. After 72 h, the amount of secreted ROS remained significantly lower (*p* < 0.0001) than the control, indicating that the antioxidant potential of the composites is maintained over time. As such, the decrease in the intensity of ROS release in both CollSS and CollSS-CQ composites, compared to the relative increase in ROS secretion in controls over time, indicates that collagen and sericin-based biomaterials have antioxidant potential, with a more significant effect in the presence of flavonoids, C and Q. Regarding the DPPH free radical scavenging activity of CQ microcapsules embedded in the material, CollSS-CQ exhibited 64.83%, compared to the scaffold without CQ, which had 13.45%, and Trolox^®^ with 81.32% (Figure 3E). Overall, the results indicate that collagen and sericin-based biomaterials determine an antioxidant effect, with a more significant effect in the presence of flavonoids C and Q.

## 4. Discussion

The field of tissue engineering is in continuous expansion to design and develop novel therapeutic strategies for wound healing and skin regeneration. Biomaterials have shown promise in the treatment of a variety of skin diseases by providing a three-dimensional in vivo-like environment to promote cell proliferation and growth [27,28]. Biomaterials based on natural polymers such as collagen and silk sericin provide suitable healing properties, as these are considered to be biocompatible and biodegradable [7,29]. Recently, multiple studies reported the use of phytocompounds, including flavonoids, in the process of tissue regeneration, given their significant antioxidant, anti-inflammatory, and immunomodulatory properties [30]. However, their therapeutic applications are limited due to insolubility in water and poor availability. These limitations can be overcome by developing innovative systems of delivery. For this purpose, we designed and developed a novel biohybrid, a natural polymeric-based composite (collagen and sericin) enriched with microcapsules containing two flavonoids, specifically curcumin and/or quercetin, to serve as a scaffold for skin tissue regeneration applications. 

Quercetin is the most active flavonoid, a polyphenol with five hydroxyl groups, which has the following peaks in the FT-IR spectrum: C=O at 1670 cm^−1^, aromatic C–H at 1614 cm^−1^, C–OH deformation at 1244 cm^−1^ and C–OH stretching at 1164 cm^−1^ [31]. Curcumin is also a flavonoid that has three reactive functional groups: one diketone moiety and two phenolic groups [32]. The FT-IR spectrum of curcumin shows a stretching band at 3510 cm^−1^ for phenolic OH groups and stretching vibrations at 1627 cm^−1^ and 1597 cm^−1^ due to C=O and C=C, respectively [33].

The FT-IR spectra of CollSS-based scaffolds containing microcapsules do not differ significantly from one another, indicating that the microcapsules maintained their integrity during the freeze-drying process and that the active ingredients, quercetin and curcumin, were not released during the obtaining procedure.

During water uptake, the spongious scaffolds start to swell slowly, reaching equilibrium after 72 h. The highest amount of water was absorbed by CollSS, reaching 40%, while the scaffolds enriched with microcapsules (CollSS-C, CollSS-Q, and CollSS-CQ) absorbed less water, having a denser structure due to their presence. Another reason for this behavior could be the hydrophobic character of both curcumin and quercetin, present in the microcapsules, which start to swell in the presence of water. The CollSS-C sponge uptakes the smallest amount of water from the first hour (19.07 g/g) to equilibrium (22.73 g/g). Taking into account that between CollSS-C and CollSS-Q there is no other difference than the encapsulated active substance, the higher amount of water uptake for CollSS-Q (26.70 to 28.66 g/g) is likely due to the fact that curcumin is more hydrophobic than quercetin. The combination of the two flavonoids in the same composite (CollSS-CQ) has more hydrophilic properties than their single use (CollSS-C and CollSS-Q), with a water uptake from 30.06 to 33.19 g/g. This behavior could be the effect of non-covalent interaction between curcumin and quercetin through hydrogen bonding or stacking interactions. 

The enzymatic degradation using collagenase revealed that, in the first three days, the scaffolds are swollen, after which they start to degrade. This is why Figure 1C shows degradation starting after 96 h. Since both curcumin and quercetin are inhibitors of collagenase [34,35,36], the samples containing these flavonoids, CollSS-C, CollSS-Q, and CollSS-CQ, degraded very slowly compared with the control one (CollSS). After six days, the simple CollSS scaffold degraded almost completely (approximately 96%), while CollSS-C degraded 37.16%, CollSS-Q 57.44%, and CollSS-CQ 15.83%. Unlike the water uptake, the scaffold enriched with both bioflavonoids, specifically CollSS-CQ, presents a synergism between curcumin and quercetin, being a stronger inhibitor of collagenase, suggesting the protective role offered to the wound dressing during the healing process. The pore size of collagen dressing is responsible for in vivo exudate absorbed [37], and it was reported that an average pore size in the range of 100–200 µm is recommended for skin tissue engineering [38,39,40]. O’Brien et al. showed a mean pore size between 95.9 and 150.5 µm for collagen–glycosaminoglicans [41]. Furthermore, the SEM images revealed the spongious structure of all tested materials, with well-defined interconnected pores and interspersed microcapsules for the CollSS-based scaffolds enriched with C and/or Q, with 50–100 µm, appropriate for potential wound healing applications. 

Biocompatibility and cytotoxicity assays (MTT, LDH, and Live/Dead) revealed an overall suitable biocompatibility of the developed composites. Curcumin and quercetin had a positive impact on cell proliferation and viability rate, especially when incorporated in the same material (CollSS-CQ). Moreover, no significant levels of cytotoxicity were observed when cells were put in contact with the materials, indicating that both curcumin and quercetin do not influence HaCaT behavior and metabolic activity. Interestingly, the MTT assay revealed that the viability of normal keratinocytes was significantly increased after 7 days of in vitro cell culture when compared to the material devoid of flavonoids or containing either one of the compounds. Hence, curcumin and quercetin present dual proliferative-enhancing properties when combined in the same composite. Simultaneously, images obtained from confocal microscopy further demonstrated an enhanced proliferation of cells at 7 days post-seeding. In the case of the material not supplemented with any compound, Live/Dead images obtained at 3 and 7 days after seeding revealed a suitable live–dead cell ratio; however, the materials enriched with either one of the flavonoids (CollSS-C and CollSS-Q) enhanced cell viability and proliferation, the best result being obtained for the material with dual activity (CollSS-CQ), results that were also confirmed through fluorescence quantification. Other studies showed that curcumin promotes tissue remodeling and granulation tissue formation in addition to collagen deposition and re-epithelialization [42,43]. Silk sericin in wound dressings also supports cell viability and proliferation, demonstrating suitable compatibility when tested in a mouse full-thickness cutaneous wound model [44]. 

Regarding the cytoskeleton development of keratinocytes in contact with the CollSS-CQ biohybrid, cytoskeleton architecture governed by actin-associated proteins was investigated. The obtained results revealed an interesting cellular behavior on the 3D composite. In the presence of both flavonoids, curcumin, and quercetin, confocal microscopy images revealed the formation of an elongated morphology and a relatively parallel distribution of F-actin filaments, indicating the changes in cell morphology. Such cellular behavior is also due to the presence of silk sericin, known as sticky protein, in the scaffold composition. In vitro studies showed that skin constructs containing silk protein can enhance keratinocytes and fibroblasts adhesion and proliferation, suggesting it is a possible component for wound healing [45]. Similar results were also reported during the crosslinking of a silk sericin-containing hydrogel. After 7 days of culture, these hydrogels promoted cell adhesion and demonstrated suitable cell colonization [46]. Enhanced migration of keratinocytes (HaCaT) and adhesion of fibroblasts were observed when investigating a freeze-dried dressing containing human-like collagen and EGF as a possible wound healing composite [47]. These observations indicate that collagen and sericin-based materials have a beneficial role in cellular behavior and mechanical properties. Also, they provide a suitable environment for these cells to proliferate, grow, and attach to the substrate. 

The effect of these composites on markers involved in apoptosis was investigated in order to assess cell survival. Over the years, numerous studies have reported the use of curcumin and quercetin as natural anti-apoptotic agents in tissue healing and regeneration [48,49,50]. The anti-apoptotic effect of quercetin was reported previously in LPS-stimulated oral mucosal keratinocytes by significantly decreasing the expression of *Bax* and increasing the expression of *Bcl-2* in a dose-dependent manner [51]. Curcumin was also shown to protect keratinocytes from apoptosis and mitochondrial damage by inhibiting *Caspase-3* and *Caspase-9* activation, in addition to increasing ERK phosphorylation [52]. Other studies reported that these flavonoids may present a pro-apoptotic activity by inducing the cell death of keratinocytes [53,54]; however, our data were consistent with the literature stating that curcumin and quercetin present an anti-apoptotic potential. In our study, we exposed the normal keratinocyte cells to three different composites supplemented with C/Q (CollSS-C, CollSS-Q, and CollSS-CQ), and we compared the obtained results to the simple composite devoid of flavonoids (CollSS). At the cellular level, it was revealed that the gene expression of the anti-apoptotic marker *Bcl-2* was significantly upregulated, while the pro-apoptotic markers *Bax* and *Caspase-3* were downregulated in the case of CollSS-based materials supplemented with flavonoids, compared to the simple composite. Moreover, the protein expression of Bax significantly increased in the presence of C and/or Q (~10× times) when compared to CollSS material. These results further validate the anti-apoptotic activity of both flavonoids. 

In addition to their anti-apoptotic properties, curcumin and quercetin are strong anti-inflammatory and antioxidant compounds. Quercetin has the ability to inhibit lipid peroxidation by scavenging free radicals and binding to metal ions [55]. It can also block the activity of multiple compounds involved in pro-inflammatory signaling pathways, such as the Mitogen-Activated Protein Kinases (MAPK) and Nuclear Factor kappa B (NFκB) pathways [56]. Curcumin can downregulate NFκB activity by binding to Toll-like receptors (TLRs). It also has the potential to regulate downstream MAPK and Activator Protein 1 (AP-1) pathways, thereby decreasing the release of pro-inflammatory mediators, such as Interleukin-1 (IL-1) and Tumor Necrosis Factor-α (TNF-α) [57]. In this context, we evaluated the anti-inflammatory properties of these compounds in the biohybrid encapsulating both flavonoids. Gene expression of pro-inflammatory cytokines IL-6 and TNF-α in activated macrophages was investigated 48 h post-seeding. IL-6 and TNF-α expression was significantly downregulated in collagen and sericin-based composite supplemented with both curcumin and quercetin in comparison to the simple composite devoid of flavonoids and to the positive control. These data were further verified and were shown to be consistent with the results obtained following analysis of IL-6 and TNF-α protein expression through both immunofluorescence and ELISA assay. Confocal microscopy images indicated a significant reduction in pro-inflammatory cytokines expression in the presence of CollSS-CQ biohybrid, correlated with a decrease in media-soluble pro-inflammatory cytokines levels. Data obtained from the study conducted by Kant et al. (2020) showed that quercetin accelerated wound repair by modulating the expression of various cytokines and growth factors such as IL-10, TNF-α, TGF-β1, VEGF, and PCNA [58]. It was also reported that the topical application of quercetin enhances wound closure and regeneration in diabetic rats by increasing the expression of IL-10, VEGF, and TGF-β1 and decreasing the expression of TNF-α, IL-1β, and MMP-9 [59]. In vivo experiments showed that micro/nanofibrous scaffolds with curcumin as ligand significantly reduced the acute inflammation stage and promoted collagen deposition during wound repair, providing a new approach for chronic wound treatment [60].

As for the antioxidant properties of these flavonoids, the same composite (CollSS-CQ) was tested to evaluate its antioxidant potential. There was a significant decrease in the release of ROS 48 h post-seeding in the case of the material enriched with flavonoids compared to simple CollSS composite and positive control, as well as a high percentage of DPPH free radical scavenging activity (~65%). It was concluded that the simultaneous use of curcumin and quercetin promotes a higher free radical scavenging activity. This effect was also reported during an in vitro study to evaluate the effect of combining curcumin and quercetin on wound healing and dermal fibroblast cell migration. In the study conducted by Chittasupho et al. (2021), results obtained from ABTS assay, which is a method that measures the scavenging capacity of antioxidants in the aqueous phase as compared with a Trolox (water-soluble vitamin E analog) standard revealed that a mixture of quercetin and curcumin (at a ratio of 3:1) was the most suitable formulation for wound healing application due to its high antioxidant capacity [61]. In vivo studies also showed that curcumin can improve the wound healing mechanism by decreasing ROS levels as a function of its phenolic hydroxyl group [62]. In conclusion, it can be stated that CollSS-based biohybrids show anti-inflammatory and antioxidant potentials, this effect being much more prominent in the case of the biohybrid supplemented with both flavonoids (CQ).

Based on the literature, the novelty of this study is highlighted by the combination of both flavonoids at the level of a single composite. Hence, considering the abovementioned properties of these flavonoids, we aim in the future to develop in vivo models to investigate the effect of these biohybrids upon implantation. Also, the development of a flavonoid-enriched composite intended for gradual delivery and release of compounds could represent a non-invasive therapeutic strategy for tissue regeneration and wound healing applications in patients suffering from chronic wounds. 

## 5. Conclusions

The effect of innovative polymeric microcapsules enriched with bioflavonoids, specifically curcumin and/or quercetin, incorporated into a composite three-dimensional material was evaluated for potential applications in wound healing and cutaneous regeneration. The scaffolds were characterized by FT-IR spectroscopy, water uptake, in vitro collagenase degradation, and SEM microscopy, while the biological properties of these composites were reported in terms of biocompatibility, cell adhesion, anti-inflammatory, and antioxidant effects. All scaffolds presented porous structure, a desirable trait for tissue regeneration, while better results in regards to withstanding degradation were obtained for CollSS-CQ due to the collagenase inhibition of both flavonoids. The enrichment of CollSS-based materials with curcumin and/or quercetin favored cellular adhesion and suitable interaction with the substrate, as revealed by confocal microscopy images, while keratinocytes placed in contact with the tested materials showed overall suitable viability and enhanced proliferation, especially in the case of the composite supplemented with both flavonoids. Moreover, the ability of these materials to support cell survival was evaluated at the gene and protein level by assessing the expression of markers involved in apoptosis. Following the exposure of keratinocytes to biomaterials enriched with C and/or Q, a significant decrease in the expression of the pro-apoptotic markers was registered, suggesting the positive effect of these flavonoids on cell survival and proliferation. In addition to that, useful traits for prospective wound dressings, such as anti-inflammatory and antioxidant properties, were investigated for the biohybrid enriched with both flavonoids, and it was observed that the tested composite displayed significant anti-inflammatory and antioxidant potential when compared to the simple CollSS material, indicated by a decrease in the gene and protein expression of inflammatory markers, as well as a decline in ROS production and increase in DPPH activity. Thus, it can be suggested that CollSS-based materials enriched with C/Q could act as potential therapeutic scaffolds for wound healing applications; however, further investigation regarding the effect of these materials is needed.

## Data Availability

The original contributions presented in the study are included in the article/supplementary material, further inquiries can be directed to the corresponding author.

## Supplementary Materials

The following supporting information can be downloaded at: https://www.mdpi.com/article/10.3390/polym16121627/s1, Figure S1: Freeze-drying chart for CollSS scaffolds; Table S1: Composition of CollSS gels with embedded C and/or Q microcapsules; Table S2: Specific primer sequences for the investigated genes.
