# Supplementary material for: Development and Investigation of an Innovative 3D Biohybrid Based on Collagen and Silk Sericin Enriched with Flavonoids for Potential Wound Healing Applications
Source: Polymers (Basel). 2024 Jun 8;16(12):1627. doi: 10.3390/polym16121627 (PMC11207284; doi:10.3390/polym16121627)
Supplement: Supplementary file 1 [file polymers-16-01627-s001.zip › polymers-3011035-supplementary.pdf]

Table S1. Composition of CollSS gels with embedded C and/or Q microcapsules

Sample code	Collagen*, %	Sericin**, %	Curcumin microcapsules*, %		Quercetin microcapsules*,%	
CollSS	1.5	0.4	0	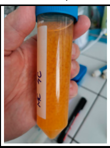	0	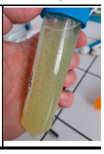
CollSS-C	1.5	0.4	30		0	
CollSS-Q	1.5	0.4	0		30	
CollSS-CQ	1.5	0.4	30		30	

* Reported to 100 ml collagen gel
**reported to collagen dry substance

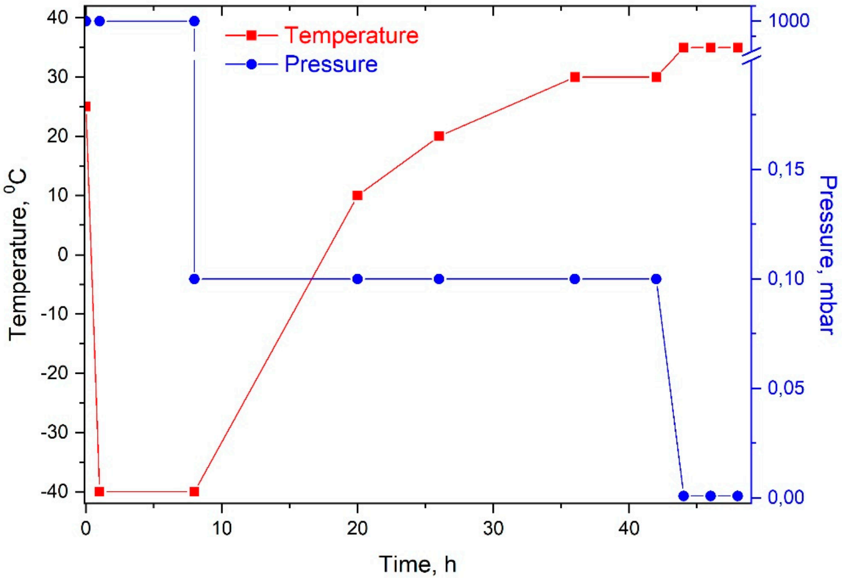

Figure S1. Freeze-drying chart for CollSS scaffolds

Table S2. Specific primer sequences for the investigated genes

**Primers**	**Type**	**Specific primer sequences**
Human Bax	*Forward*	*TTTGCTTCAGGGTTTCATCCA*
	Reverse	*GAGACACTCGCTCAGCTTCTTG*
Human Bcl-2	*Forward*	*GTGCCTGCTTTTAGGAGACCGA*
	Reverse	*GAGACCACACTGCCCTGTTGATC*
Human Caspase-3	*Forward*	*GTAGAAGTCTAACTGGAAAACCCAA*
	Reverse	*CATGTCATCATCAACACCACTGTCT*
Human TNFα	*Forward*	*CTCTTCTGCCTGCTGCACTTTG*
	Reverse	*ATGGGCTACAGGCTTGTCACTC*
Human IL-6	*Forward*	*AGACAGCCACTCACCTCTTCAG*
	Reverse	*TTCTGCCAGTGCCTCTTTGCTG*
Human GAPDH	*Forward*	*TTTGCTTCAGGGTTTCATCCA*
	Reverse	*GAGACACTCGCTCAGCTTCTTG*
